# Artificial intelligence-driven precision immunogenetics for cancer stem cell-targeted CAR immunotherapy

**DOI:** 10.1186/s43046-026-00422-2

**Published:** 2026-11-05

**Authors:** Visula Abeysuriya, Sachintha N. Alwis, Vasitha Abeysuriya

**Affiliations:** 1 University of Colombo, Institute of Biochemistry, Molecular Biology and Biotechnology, Colombo, Sri Lanka; 2 Department of Biomedical Science, School of Medical Sciences, International Institute of Health Sciences (IIHS – Multiversity), Wattala, Sri Lanka; 3 University of Kelaniya, Faculty of Medicine, Kelaniya, Sri Lanka

**Keywords:** Artificial intelligence, Cancer stem cells, CAR immunotherapy, Next-generation CAR therapies, Precision immunogenetics, Therapeutic resistance, Tumour microenvironment

## Abstract

Cancer stem cells (CSCs) are a major contributor to therapeutic resistance, tumour recurrence, and disease progression because of their self-renewal capacity, phenotypic plasticity, immune evasion, and ability to persist after treatment. Chimeric antigen receptor (CAR)-based immunotherapy has transformed the treatment of several hematological malignancies, but effective targeting of CSC-driven disease, especially solid tumour, remains challenging. Antigen heterogeneity and loss, CSC plasticity, CAR-cell dysfunction, metabolic constraints, and an immunosuppressive tumour microenvironment (TME) can limit long-term therapeutic responses. CSC-associated antigens such as CD133, CD44, EpCAM, HER2, CSPG4, ALDH, and EGFRvIII have been investigated as CAR targets, although their heterogeneous and context-dependent expression raises important concerns regarding CSC specificity and on-target/off-tumour toxicity. Artificial intelligence (AI) and computational approaches provide opportunities to integrate genomic, transcriptomic, proteomic, spatial, and clinical data for CSC-associated antigen discovery, biomarker identification, CAR design, patient stratification, and toxicity prediction. However, the evidence supporting these applications varies considerably, ranging from computational and preclinical studies to retrospective clinical modelling, while prospective clinical validation of AI-guided CSC-targeted CAR therapy remains limited. Similarly, next-generation platforms, including CAR-natural killer (CAR-NK) cells, CAR-macrophages (CAR-M), gamma-delta CAR-T cells, and CAR-invariant natural killer T (CAR-iNKT) cells, remain at different stages of translational development. This review critically examines CSC biology, mechanisms of resistance to CAR-based therapy, emerging CAR platforms, and the potential role of AI in precision immunogenetics. Particular emphasis is placed on differentiating CSC-intrinsic, CAR-cell-intrinsic, and TME-mediated resistance and on defining the evidence maturity of AI applications. Finally, current challenges in data availability, tumour heterogeneity, model bias, external validation, interpretability, safety, and prospective clinical translation are discussed, together with priorities for developing clinically validated AI-enabled CSC-targeted CAR therapies.

## Introduction

Cancer remains a major cause of morbidity and mortality despite advances in surgery, chemotherapy, radiotherapy, targeted therapy, and immunotherapy. A major limitation of long-term tumour control is the persistence of cancer stem cells (CSCs), which possess self-renewal, tumour-initiating capacity, phenotypic plasticity, and resistance to therapeutic stress. CSCs have therefore been implicated in tumour progression, metastasis, treatment resistance, and disease recurrence across solid and haematological malignancies [1–5]. Importantly, CSC resistance should not be viewed as a fixed phenotype. Stemness-associated transcriptional and epigenetic programmes, cellular plasticity, and treatment-induced state transitions can allow tumour cells to acquire or lose CSC-like characteristics during disease progression and treatment. This dynamic behaviour complicates the identification of stable therapeutic targets.

Chimeric antigen receptor (CAR) immunotherapy helps immune cells to recognize cell-surface antigens independently of major histocompatibility complex (MHC) presentation and has produced substantial clinical benefit, especially in haematological malignancies [6–8]. The success of CAR-T-cell therapy has subsequently driven the development of alternative platforms, including CAR-natural killer (CAR-NK) cells, CAR-macrophages (CAR-M), and other engineered immune-cell approaches [8–14]. However, these platforms are not at equivalent stages of development. CAR-T cells have the strongest clinical evidence, whereas many alternative platforms remain in early clinical or preclinical development. Their potential advantages, including different mechanisms of tumour recognition, trafficking, persistence, and interaction with the tumour microenvironment (TME), therefore require assessment according to their individual biological and translational maturity.

Several CSC-associated antigens, including CD133, CD44, EpCAM, HER2, CSPG4, ALDH, and EGFRvIII, have been investigated as CAR targets in different tumour types [6–11]. However, CSC-associated expression does not necessarily indicate CSC specificity. Expression may vary between tumour types, patients, tumour regions, and cellular states, while some proposed CSC markers are also expressed by normal tissues or non-CSC tumour populations. Consequently, antigen heterogeneity, antigen loss, phenotypic plasticity, and potential on-target/off-tumour toxicity remain major barriers to long-term CSC-directed CAR therapy [12–19]. These limitations are particularly important in solid tumour, where restricted trafficking, stromal barriers, hypoxia, metabolic stress, and immunosuppressive immune populations further reduce CAR-cell activity.

Resistance therefore arises from multiple interacting compartments rather than from CSCs alone. CSC-intrinsic mechanisms include stemness-associated signalling, phenotypic plasticity, and adaptive changes in antigen expression. CAR-cell-intrinsic mechanisms include impaired persistence, chronic antigen stimulation, metabolic dysfunction, and T-cell exhaustion. In parallel, the TME can restrict immune-cell infiltration and function through cancer-associated fibroblasts (CAFs), extracellular-matrix remodeling, hypoxia, suppressive myeloid populations, regulatory T cells, and inhibitory signalling pathways. Distinguishing these mechanisms is essential for identifying whether a therapeutic strategy should modify the CSC target, the CAR-cell state, or the surrounding microenvironment.

Artificial intelligence (AI) and computational approaches provide opportunities to integrate genomic, transcriptomic, proteomic, single-cell, spatial, imaging, and clinical data for biomarker discovery, patient stratification, antigen prioritization, and CAR engineering [18, 20–23]. The translational maturity of these applications varies substantially. Computational target prediction, preclinical modelling, retrospective clinical prediction, and prospective clinical validation represent distinct evidence levels and should not be considered equivalent. Therefore, prediction of immunotherapy response does not demonstrate that an AI model has prospectively designed an effective CSC-targeted CAR product.

This review therefore evaluates CSC-targeted CAR immunotherapy according to three interconnected domains: CSC-intrinsic biology, CAR-cell-intrinsic function, and TME-mediated resistance. It further examines AI applications according to their evidence and validation stage, with emphasis on the distinction between computational discovery, experimental or preclinical validation, retrospective clinical modelling, and prospective clinical utility. The review also considers the differences in biological function, manufacturing, clinical maturity, and translational potential among CAR-T, CAR-NK, CAR-M, gamma-delta CAR-T, and CAR-invariant natural killer T (CAR-iNKT) platforms. Finally, the major barriers to clinical implementation, including CSC-specific dataset scarcity, tumour heterogeneity, model bias, batch effects, external validation, interpretability, safety, and regulatory accountability are considered in the context of developing clinically validated AI-enabled CSC-targeted CAR therapies.

## CAR platforms for CSC-targeted immunotherapy

CSCs are relevant targets for CAR-based therapy because their persistence contributes to tumour regeneration, treatment resistance, and recurrence. CAR platforms provide MHC-independent antigen recognition, but their effectiveness against CSCs is constrained by antigen heterogeneity, CSC plasticity, limited persistence, and TME-mediated suppression [11, 24–28]. The available CAR platforms differ substantially in clinical maturity, biological function, and manufacturing feasibility. CAR-T cells have the strongest clinical evidence, whereas CAR-NK, CAR-M, gamma-delta CAR-T, CAR-iNKT, and CAR-engineered neutrophils remain at earlier stages of development.

### CAR-T cells

CAR-T cells remain the most clinically advanced CAR platform, with established efficacy in several haematological malignancies. Their MHC-independent recognition is advantageous because reduced MHC-I expression can contribute to immune escape [29–36]. CSC-associated targets investigated for CAR-T therapy include CD133, EpCAM, CD44v6, CD123, HER2, and EGFR variants. However, targeting a single antigen may be insufficient because antigen loss, heterogeneous expression, and lineage plasticity can permit survival of residual tumour populations [30, 36–38].

Persistent antigen exposure, inhibitory signalling, and metabolic stress can also impair CAR-T-cell function. Importantly, increased expression of inhibitory receptors should not automatically be equated with irreversible terminal exhaustion; exhaustion represents a broader functional and transcriptional state involving persistent stimulation, altered differentiation, metabolic dysfunction, and reduced effector capacity [39, 40]. Accordingly, next-generation CAR designs may delay or reduce dysfunction, but they should not be assumed to completely prevent exhaustion.

**Fig. 1:**
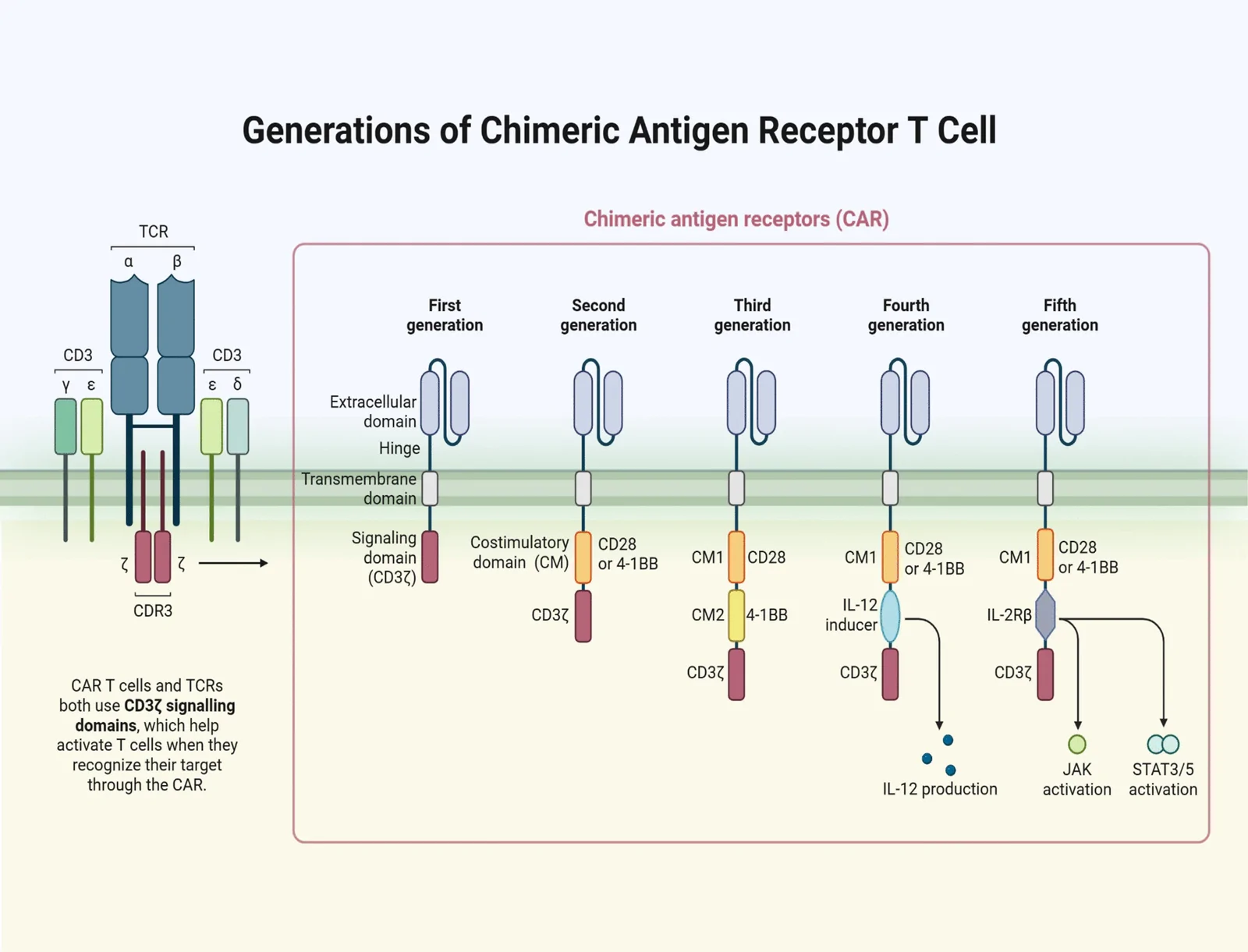
Evolution of chimeric antigen receptor (CAR) generations. Schematic illustration of the structural evolution of CAR constructs from first- to fifth-generation designs. Successive generations incorporate additional intracellular signalling and co-stimulatory domains to enhance T-cell activation, proliferation, persistence, cytokine production, and anti-tumor efficacy. Fifth-generation CARs further integrate cytokine receptor signalling to activate downstream JAK–STAT pathways and improve therapeutic function. Created with BioRender.com

Dual-target, tandem, and logic-gated CAR designs may reduce antigen escape compared with single-antigen targeting, but increased targeting breadth does not eliminate the risks associated with heterogeneous antigen expression or normal-tissue expression [20, 24, 25, 27]. Thus, CAR-T therapy currently provides the strongest platform for CSC-directed clinical translation, but effective CSC eradication remains dependent on appropriate target selection and control of tumour and CAR-cell resistance mechanisms. The structural evolution of CAR constructs across successive generations, including the incorporation of additional co-stimulatory and cytokine-signalling domains, is illustrated in Fig. 1.

### CAR-NK cells

CAR-NK cells combine CAR-dependent antigen recognition with innate cytotoxic mechanisms and may offer advantages in allogeneic manufacturing because of a lower risk of graft-versus-host disease (GVHD) than conventional allogeneic T-cell approaches [21]. Their potential advantages include reduced risks of cytokine release syndrome (CRS) and neurotoxicity and the possibility of developing standardized off-the-shelf products [21].

However, limited in vivo persistence remains a major limitation. Strategies such as IL-15 expression and CISH deletion have been investigated to improve CAR-NK survival, metabolic fitness, and cytotoxic activity [41, 42]. NK-cell activity is also shaped by the TME: cytokine and growth-factor networks can reprogrammed NK cells toward dysfunctional or immunoregulatory states, so innate cytotoxicity should not be assumed to remain intact in every tumour context. This functional plasticity is relevant when designing CAR-NK products and combination strategies. Although CAR-NK cells may offer advantages in safety and manufacturing, long-term CSC eradication remains less clinically established than CAR-T therapy.

### CAR-macrophages

CAR-M cells provide a distinct biological approach to solid tumour because macrophages can infiltrate tumour tissue, phagocytose malignant cells, modify the TME, and contribute to antigen presentation [43–45]. These properties may be especially relevant to CSC-rich solid tumour in which poor CAR-T infiltration and stromal barriers limit therapeutic activity.

However, CAR-M therapy remains substantially less clinically validated than CAR-T therapy. Its potential should therefore be considered in the context of tumour infiltration, phagocytic activity, TME remodeling, and antigen presentation rather than as an established replacement for CAR-T cells [22].

### Gamma-delta CAR-T cells

Gamma-delta CAR-T cells combine CAR-mediated antigen recognition with the intrinsic tumour-recognition properties of gamma-delta T cells, which can recognize cellular stress-associated signals without conventional peptide–MHC restriction. This may broaden tumour recognition and provide an additional route to target heterogeneous disease, although gamma-delta subsets differ in phenotype, tissue distribution, and functional behaviour. Reviews of CAR-gamma-delta T-cell development describe encouraging preclinical findings but emphasize that manufacturing, persistence, functional consistency, and clinical evidence remain important limitations [23, 46]. Their efficacy against CSC populations specifically has not been established clinically.

### CAR-iNKT cells

CAR-invariant natural killer T (CAR-iNKT/CAR-NKT) cells combine CAR-dependent antigen recognition with invariant T-cell receptor (TCR) recognition and innate-like effector functions. Unlike conventional αβ T cells, iNKT cells have a restricted invariant TCR and can recognize CD1d-presented lipid antigens; this biology may permit recognition of selected immunosuppressive CD1d-positive cells in the tumour microenvironment (TME), in addition to CAR-directed tumour killing. Recent preclinical studies have demonstrated allogeneic, hematopoietic stem and progenitor cell (HSPC)-derived CAR-NKT platforms directed against ovarian cancer, CD70-positive renal cell carcinoma, mesothelin-positive triple-negative breast cancer, and EGFRvIII-positive glioblastoma. Across these models, tumour killing involved CAR- and/or NK-receptor-mediated activity, while TCR-mediated recognition of CD1d-positive cells provided a potential means of modifying the TME. HSPC-derived approaches also offer a scalable route to off-the-shelf manufacture and may reduce alloreactivity and graft-versus-host disease risk compared with conventional allogeneic αβ T cells [29–32, 37]. These findings are strong but remain predominantly preclinical; the cited studies do not establish clinical efficacy or long-term CSC eradication. The biological rationale should therefore be distinguished from demonstrated clinical benefit.

The evidence base is evolving rapidly, but the recent studies primarily establish platform feasibility and antitumor activity in experimental models rather than mature clinical outcomes. Table 1 therefore differentiate early translational development from established clinical validation and does not classify these HSPC-derived platforms as clinically proven therapies [29–32, 37].

**Table 1:** Comparison of CAR platforms for CSC-targeted immunotherapy

CAR platform	Biological advantage	CSC relevance	Major limitations	Development stage	Clinical evidence	Manufacturing/allogeneic potential	Representative references
CAR-T cells	Potent antigen-specific cytotoxicity; established persistence and expansion	Most extensively investigated platform for targeting CSC-associated antigens including CD133, EpCAM, CD44v6, CD123, HER2 and EGFR variants	Antigen escape; T-cell exhaustion; CRS; limited trafficking and efficacy in many solid tumour	Clinically established	Strongest clinical evidence, particularly in hematological malignancies; CSC-specific efficacy remains less established	Autologous manufacturing is complex and time-consuming; allogeneic approaches remain under development	[10, 20–28, 39, 40]
CAR-NK cells	CAR-mediated targeting combined with innate cytotoxicity; potential for reduced GVHD and CRS; MHC-independent killing	May improve recognition of heterogeneous tumour populations and provide an off-the-shelf approach for CSC-targeted therapy	Limited persistence and expansion; optimization of metabolic fitness remains necessary	Early clinical/translational	Early clinical evidence; long-term CSC-specific clinical benefit remains insufficiently established	Strong potential for allogeneic and off-the-shelf manufacturing	[21, 22, 47]
CAR-macrophages (CAR-M)	Efficient tumour infiltration, phagocytosis, TME remodeling and antigen presentation	Particularly relevant to solid tumour and CSC niches where poor immune-cell infiltration and immunosuppression limit CAR-T activity	Limited clinical validation; macrophage functional state and persistence require optimization	Early clinical/translational	Limited early clinical evidence; CSC-specific clinical efficacy remains unestablished	Potential for engineered myeloid-cell manufacturing; allogeneic feasibility requires further validation	[41, 42, 48]
gamma-delta CAR-T cells	CAR targeting combined with intrinsic, MHC-independent tumour recognition	Potentially broadens tumour recognition; CSC-specific efficacy remains unproven	Heterogeneous gamma-delta subsets; variable expansion, persistence and functional consistency; limited clinical evidence	Preclinical/early translational	No established clinical evidence for CSC eradication; broader gamma-delta-cell clinical experience should not be conflated with CAR-gamma-delta efficacy	Potential for allogeneic manufacture; protocols and product consistency require validation	[30, 32, 46]
CAR-iNKT cells	CAR-mediated tumour recognition plus invariant TCR- and NK-receptor-associated functions	Potential dual activity against tumour cells and selected CD1d-positive immunosuppressive TME cells; CSC-specific benefit unproven	Evidence is predominantly preclinical; antigen context, TME interactions, persistence, differentiation and product consistency require further evaluation	Preclinical/early translational	Recent HSPC-derived allogeneic studies show preclinical antitumor activity in ovarian cancer, RCC, TNBC and GBM models; mature clinical efficacy data are not established	Cord-blood CD34 + HSPC-derived platforms offer a scalable off-the-shelf strategy; low alloreactivity/GVHD risk is promising but requires clinical validation	[29–32, 37]
CAR-engineered neutrophils	Rapid migration toward inflamed tumour tissues and potential for enhanced tumour infiltration	Potentially useful for poorly accessible solid-tumour niches, including CSC-containing regions	Short lifespan; predominantly preclinical evidence; CSC-specific activity remains unclear	Predominantly preclinical	No established clinical evidence for CSC eradication	Manufacturing and engineering approaches remain experimental	[33]

Development stages are presented comparatively to distinguish the established clinical position of CAR-T cells from the earlier translational status of alternative CAR platforms. The presence of a platform in early clinical development does not establish clinical efficacy against CSCs

*Abbreviations- CAR* chimeric antigen receptor, *CSC* cancer stem cell, *CAR-T* CAR T cell, *CAR-NK* CAR natural killer cell, *CAR-M* CAR macrophage, *iNKT* invariant natural killer T cell, *TME* tumour microenvironment, *GVHD* graft-versus-host disease, *CRS* cytokine release syndrome, *MHC* major histocompatibility complex

### CAR-engineered neutrophils

CAR-engineered neutrophils represent an emerging platform with rapid migration toward inflamed tumour tissues [33]. Their potential advantage is therefore primarily related to tumour trafficking rather than established CSC-specific cytotoxicity. Evidence emphasizing their application in CSC-targeted therapy remains largely preclinical [34, 35].

### Comparative translational perspective

**Fig. 2:**
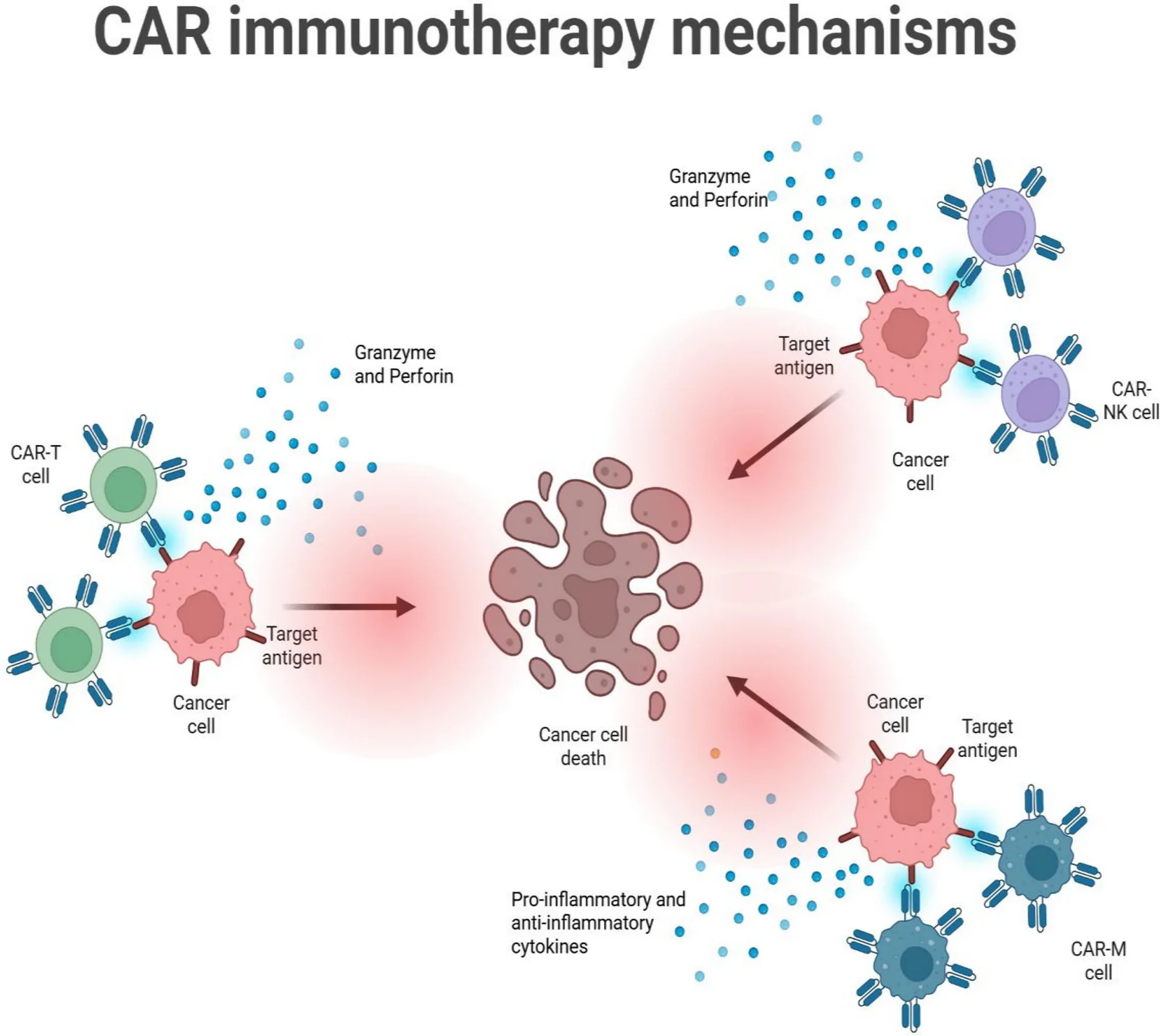
Mechanisms of tumour cell elimination by CAR-engineered immune-cell platforms. Schematic representation of the principal mechanisms through which CAR-engineered immune cells recognize and eliminate tumour cells. CAR-T cells primarily induce tumour cell apoptosis through perforin- and granzyme-mediated cytotoxicity, CAR-NK cells combine innate and CAR-dependent cytotoxic mechanisms, and CAR-macrophages (CAR-M) promote tumour clearance through phagocytosis, cytokine secretion, and antigen presentation, collectively contributing to enhanced anti-tumor immunity. Created with BioRender.com

The major CAR platforms should therefore be distinguished by biological mechanism and development stage rather than presented as equivalent alternatives. CAR-T cells have the strongest clinical validation but remain limited by antigen escape, exhaustion, and poor activity in many solid tumors. CAR-NK cells offer potential advantages in allogeneic manufacturing and safety but remain constrained by persistence. CAR-M cells may be particularly suited to solid tumour because of their infiltration, phagocytosis, and TME-remodeling capacity, although clinical evidence remains limited. gamma-delta CAR-T and CAR-iNKT cells provide additional MHC-independent immune functions but remain in earlier clinical development, whereas CAR-engineered neutrophils are predominantly preclinical [25, 33]. Consequently, no single CAR platform currently provides a universal solution for CSC eradication. Platform -selection should instead consider CSC antigen distribution, tumour accessibility, TME characteristics, expected persistence, manufacturing requirements, allogeneic feasibility, and the specific resistance mechanism that must be addressed. A comparative assessment of these characteristics is provided in Table 1. The principal mechanisms of tumour-cell recognition and elimination by the major CAR-engineered immune-cell platforms are summarized in Fig. 2.

## Mechanisms of resistance to CAR-based therapy

**Fig. 3:**
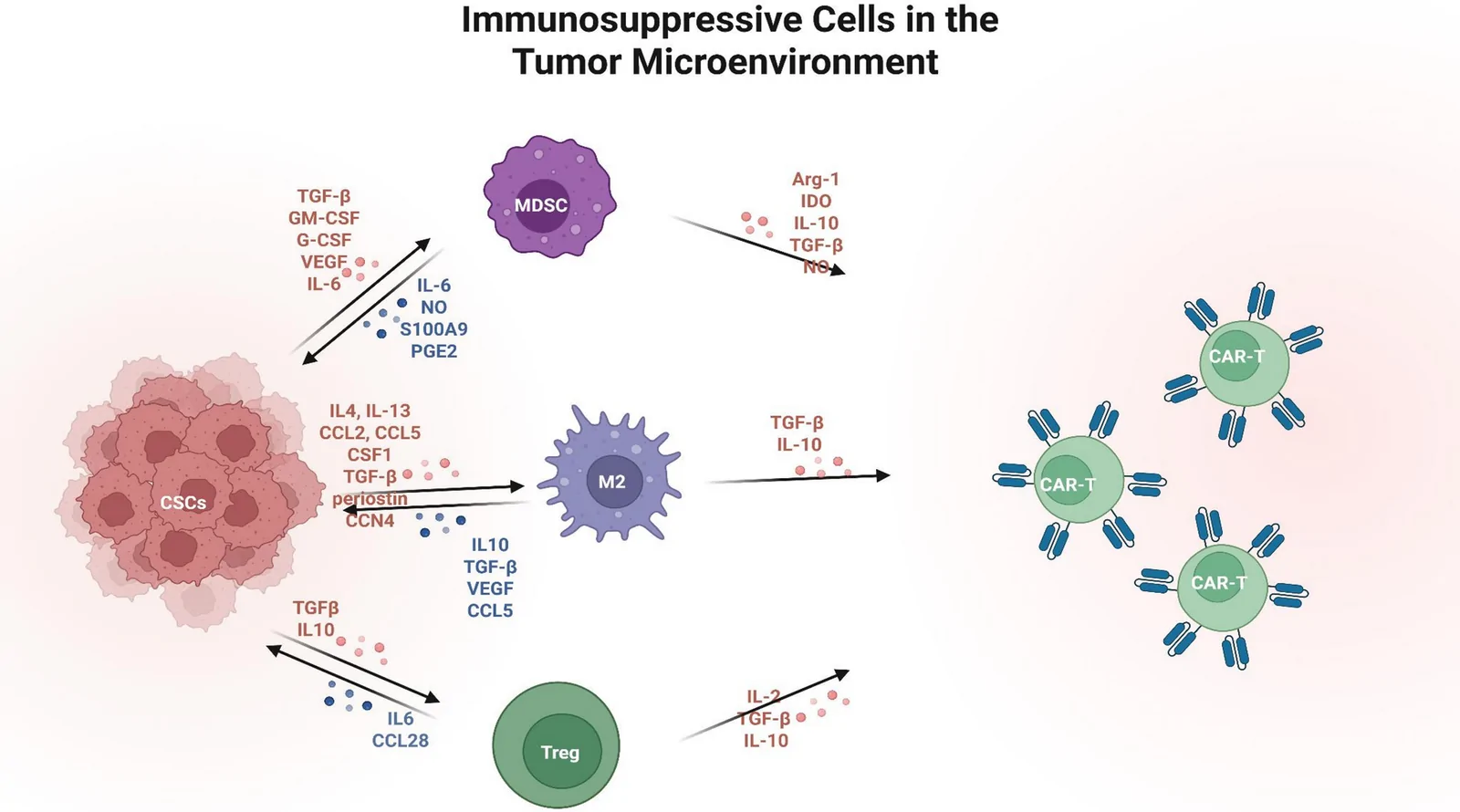
Immunosuppressive tumour microenvironment limiting CAR-T-cell function. Schematic overview of the immunosuppressive tumour microenvironment illustrating how regulatory T cells, tumour-associated macrophages, myeloid-derived suppressor cells, cancer-associated fibroblasts, inhibitory cytokines, immune checkpoint signalling, hypoxia, and metabolic dysregulation collectively impair CAR-T-cell activation, persistence, proliferation, and cytotoxic function, thereby promoting immune evasion and therapeutic resistance. Created with BioRender.com

Despite advances in CAR engineering, long-term elimination of cancer stem cells (CSCs) remains challenging because resistance is mediated by multiple interacting mechanisms rather than by a single biological defect. CSCs can modify antigen expression, alter stemness-associated states, exploit immune-regulatory pathways, and adapt to metabolic and stromal conditions during therapeutic pressure [26–28]. These mechanisms can be broadly differentiated into CSC-intrinsic resistance, CAR-cell-intrinsic dysfunction, and tumour microenvironment (TME)-mediated resistance, although substantial crosstalk occurs between these compartments. This distinction is important because each resistance category requires a different therapeutic intervention. The principal interactions among CSCs, CAR cells, CAFs, suppressive immune populations, hypoxia, metabolic constraints, and immune-regulatory pathways are summarized in Fig. 3.

### CSC-intrinsic resistance

CSC-intrinsic resistance includes antigen heterogeneity, antigen loss, stemness-associated signalling, and phenotypic plasticity. Although CAR recognition is independent of major histocompatibility complex (MHC) presentation, effective targeting still requires sufficient and stable expression of the target antigen. CSC populations are heterogeneous and can therefore contain antigen-low or antigen-negative subpopulations that survive selective immune pressure. Reduced expression of CD123 in plastic plasmacytoid dendritic cell neoplasm and sequential loss of CD19 and CD22 following CAR-T-cell therapy illustrate how antigen escape can contribute to relapse despite an initial response [20]. These observations help the development of multi-antigen, tandem, and logic-gated CAR designs rather than reliance on a single target [20, 24, 25].

CSC plasticity further complicates this strategy because stem-like characteristics are not necessarily confined to a permanently defined cellular population. Differentiated tumour cells may reacquire stem-like properties through developmental signalling networks involving Wnt/β-catenin, Notch, Hedgehog, PI3K/AKT/mTOR, and Hippo pathways [27, 28, 36, 38]. Consequently, treatment may not simply eliminate a pre-existing CSC population; it can also impose selective pressure that favors the emergence or enrichment of resistant stem-like states. This dynamic behaviour creates a distinction between static CSC markers and dynamic CSC states, with important implications for target discovery and therapeutic design.

Epigenetic and transcriptional plasticity may further modify antigen expression, differentiation state, and susceptibility to immune-mediated killing. RNA-regulatory mechanisms and treatment-induced changes in cellular state can contribute to transitions between stem-like and more differentiated phenotypes. Therefore, identification of therapeutically useful CSC targets requires assessment not only of baseline expression but also of how target expression changes during tumour evolution and CAR-mediated selection [27, 28, 36, 38].

### CAR-cell-intrinsic dysfunction and T-cell exhaustion

Resistance is also determined by the functional state of the CAR-engineered immune cell. Persistent exposure to tumour antigen can result in progressive loss of proliferation, cytokine production, cytotoxic activity, and persistence. In CAR-T cells, chronic antigen stimulation and inhibitory signalling can promote an exhausted or dysfunctional state, thereby limiting sustained tumour control [39, 40].

Importantly, expression of inhibitory receptors such as programmed cell death protein 1 (PD-1) should not automatically be equated with terminal T-cell exhaustion. Checkpoint expression may represent a reversible state of activation or dysfunction, whereas established exhaustion involves broader transcriptional, epigenetic, metabolic, and differentiation-associated changes. Persistent antigen exposure, altered cellular metabolism, mitochondrial dysfunction, and progressive differentiation can collectively reduce the capacity of CAR-T cells to proliferate and maintain cytotoxic function [41, 48].

Current engineering strategies may therefore act at different stages of this process. Checkpoint blockade or checkpoint-resistant CAR designs may partially restore function, whereas modifications aimed at improving metabolic fitness, persistence, or differentiation state may delay the development of dysfunction. However, these approaches should not be interpreted as universally preventing or reversing exhaustion. Their effectiveness depends on the intensity and duration of antigen exposure and the surrounding TME.

This limitation also provides a biological rationale for alternative CAR platforms. CAR-NK cells combine CAR-dependent recognition with innate cytotoxic mechanisms, whereas CAR-macrophages can infiltrate tumour tissue and contribute to phagocytosis and antigen presentation [21, 41, 42, 48, 49]. The principal tumour-cell elimination mechanisms of these platforms differ from conventional CAR-T-cell cytotoxicity and are summarized in Fig. 2. Alternative platforms also have platform-specific limitations, including limited persistence for CAR-NK cells and incomplete clinical validation for CAR-M cells [21, 41, 42, 48, 49].

### Immune checkpoint and innate immune evasion

Immune-regulatory pathways provide an additional layer of resistance. CSCs in several tumour contexts have been reported to exhibit increased expression of immune-regulatory molecules such as PD-L1 and CD47, although expression varies according to tumour type, cellular state, and microenvironmental conditions [31, 48, 50–57]. PD-1/PD-L1 signalling can suppress CAR-T-cell function and contribute to progressive dysfunction, whereas CD47 signalling can inhibit macrophage-mediated phagocytosis [41, 58, 59].

The therapeutic implications are therefore platform-dependent. PD-1/PD-L1 blockade may improve CAR-T-cell activity in selected settings, while interruption of CD47-mediated signalling may be particularly relevant to macrophage-based approaches. Engineered CAR-T and CAR-NK cells with locally modified checkpoint pathways have also shown enhanced anti-tumour activity in experimental models [36, 37, 41]. CD47-targeted approaches also face important translational challenges, including expression of CD47 on normal cells, potential haematological toxicity, and the need to balance macrophage activation with tolerability. Thus, CD47 is a biologically relevant innate immune checkpoint, but its blockade should not be presented as a universally effective strategy; efficacy and safety depend on tumour context and therapeutic design.

### TME-mediated resistance

The TME provides a further level of resistance by restricting CAR-cell trafficking, activation, persistence, and cytotoxicity. CSCs should not be considered passive inhabitants of this environment. CSC populations can interact bidirectionally with stromal and immune compartments through cytokines, chemokines, extracellular vesicles, and metabolic signals, thereby contributing to formation of a protective niche [41–45, 48, 50–67].

CSCs can promote recruitment or activation of myeloid-derived suppressor cells, tumour-associated macrophages, and regulatory T cells through mediators including IL-6, IL-10, TGF-β, CCL2, CCL5, VEGF, and GM-CSF [44, 45, 50–67]. These populations can suppress CAR-cell activation and persistence while promoting conditions favorable for CSC survival.

Cancer-associated fibroblasts (CAFs) represent an additional stromal barrier. CAF-mediated extracellular-matrix remodeling can increase tissue stiffness and create physical barriers to immune-cell infiltration, while stromal signalling can provide metabolic and survival support to tumour-cell populations [68–73]. Importantly, these stromal compartments can also adapt to therapeutic pressure. Thus, CAR-cell exposure may alter the cellular composition and signalling environment of the tumour, potentially selecting for resistant CSC states rather than simply encountering a pre-existing static barrier.

CSC-derived extracellular vesicles and other secreted factors can further reinforce immunosuppression by modifying immune-cell function and stromal behaviour [54–59]. The resulting network means that CAR resistance cannot be adequately explained by either CSC biology or TME suppression alone. CSCs can construct or exploit protective methods, while immune-mediated tumour selection can subsequently enrich resistant cellular states.

### Metabolic and hypoxic resistance

Metabolic adaptation represents another interconnected mechanism of resistance. Increased glycolysis, lactate accumulation, hypoxia, and altered fatty-acid metabolism can support CSC survival while impairing CAR-cell function [49, 74–82]. Lactate-rich conditions can suppress T-cell and natural killer (NK)-cell metabolism and cytotoxicity, whereas hypoxia can reduce CAR-T-cell expansion and cytokine production [49, 74–82]. Altered fatty-acid oxidation may simultaneously support CSC maintenance and increase resistance to immune-mediated apoptosis [49, 74–82].

These metabolic mechanisms are closely linked to the cellular and stromal components of the TME. Hypoxia can influence immune-cell differentiation and function, while CAFs and other stromal populations can contribute to nutrient competition and metabolic support. Therefore, metabolic resistance should be considered as part of the broader CSC–CAR–TME network rather than as an isolated pathway.

Experimental approaches targeting lactate metabolism, lipid metabolism, or hypoxia-adaptive CAR-cell function have produced encouraging preclinical findings [49, 74–82]. However, these approaches remain predominantly experimental, and their ability to produce long-term CSC eradication in patients has not yet been established.

### Integrated CSC–CAR–TME resistance network

CSC resistance emerges from continuous interaction between tumour-cell state, CAR-cell function, and the surrounding microenvironment. CSC-intrinsic plasticity can alter antigen availability; CAR-cell dysfunction can reduce immune pressure; and TME-mediated stromal, immune, and metabolic barriers can further protect resistant tumour-cell populations. Therapeutic pressure may then select for cellular states that are less susceptible to subsequent CAR-mediated elimination.

The principal interactions among CSCs, CAR cells, CAFs, suppressive immune populations, hypoxia, metabolic constraints, and immune-regulatory pathways are summarized in Fig. 3. This integrated model emphasizes that successful CSC-directed therapy will probably require simultaneous consideration of target stability, CAR-cell fitness, and the tumour ecosystem rather than optimization of antigen recognition alone.

These mechanisms provide the biological basis for an evidence-driven precision approach: dynamic CSC states and candidate antigens must first be identified, CAR-cell properties must then be experimentally optimized, and the resulting strategy must be evaluated within the relevant TME and tumour context. This framework also defines where artificial intelligence (AI) may contribute especially in identifying dynamic biomarkers, integrating heterogeneous datasets, prioritizing multi-antigen strategies, and predicting patient- or tumour-specific resistance but computational prediction alone cannot establish therapeutic efficacy [49, 74–81]. These interconnected mechanisms indicate that resistance to CAR-based therapy results from interactions among CSC state, CAR-cell function, and the surrounding TME rather than from a single resistance pathway. The principal CSC-intrinsic, CAR-cell-intrinsic, and TME-mediated mechanisms of resistance, together with their therapeutic implications, are summarized in Table 2.

**Table 2:** Mechanisms of resistance to CSC-targeted CAR immunotherapy

Resistance category	Major mechanisms	Consequences for CAR therapy	Potential therapeutic implications	Representative references
CSC-intrinsic resistance	Antigen heterogeneity and loss; stemness-associated signalling; epigenetic and transcriptional plasticity; treatment-induced state transitions; altered antigen expression	Antigen escape and persistence of resistant tumour-cell states	Multi-antigen targeting; dynamic biomarker identification; targeting stemness-associated pathways	[28, 38, 39, 48, 49]
CAR-cell-intrinsic dysfunction	Chronic antigen exposure; T-cell exhaustion; impaired proliferation and persistence; mitochondrial and metabolic dysfunction; altered differentiation states	Reduced cytotoxicity, persistence and expansion of CAR cells	Optimized CAR signalling; strategies targeting exhaustion and metabolic dysfunction; next-generation CAR platforms	[20, 21, 83]
Immune checkpoint and innate immune evasion	PD-1/PD-L1 signalling; CD47-mediated suppression of phagocytosis; other inhibitory pathways	Reduced CAR-cell activity and impaired innate immune clearance	Checkpoint blockade; CD47-targeting strategies; combination or engineered CAR approaches	[24–26, 39, 40, 44, 45, 60–66, 84, 85]
TME-mediated resistance	CAF-mediated stromal exclusion; extracellular-matrix remodeling; Treg, TAM and MDSC accumulation; suppressive cytokines and chemokines	Impaired trafficking, activation and persistence of CAR cells	TME remodeling; stromal targeting; combination immunotherapy	[28, 35, 36, 38, 41–45, 48–60, 66, 67, 74, 86]
Metabolic and hypoxic resistance	Hypoxia; lactate accumulation; altered glycolysis and fatty-acid metabolism; nutrient competition	Reduced CAR-cell proliferation, cytokine production and cytotoxicity	Metabolic intervention; hypoxia-responsive CAR designs; combination strategies	[49, 68, 75–82, 87–91]
Integrated adaptive resistance	Interaction between CSC plasticity, CAR-cell dysfunction and TME adaptation under therapeutic pressure	Selection and persistence of treatment-resistant tumour states	Longitudinal monitoring; multi-antigen strategies; combined CSC-, CAR- and TME-directed approaches	[10, 25, 26, 49]

Resistance mechanisms frequently overlap and may operate simultaneously. CSC-intrinsic mechanisms describe adaptations occurring within tumour/CSC populations; CAR-cell-intrinsic mechanisms describe dysfunction of the therapeutic immune cells; and TME-mediated mechanisms describe effects arising from stromal, immune, metabolic, and physical components of the tumour niche

## CSC-associated antigens and safety considerations

The therapeutic effectiveness of CSC-directed CAR immunotherapy depends fundamentally on the selection of antigens that are sufficiently enriched on tumour-initiating populations while remaining accessible to CAR-engineered immune cells. Several molecules, including CD133, CD44, EpCAM, CD44v6, CD123, HER2, CSPG4, ALDH, and EGFR variants, have been investigated as potential targets across different malignancies [7–11, 20, 24, 25, 27]. However, the designation of an antigen as a CSC-associated marker should not be interpreted as evidence that it is exclusively expressed by CSCs.

### CSC-associated versus CSC-specific antigens

An important conceptual distinction is that CSC-associated expression does not necessarily imply CSC specificity. A marker may enrich for a CSC population experimentally without providing sufficient specificity for therapeutic CAR recognition. CSC-associated antigens can also be expressed by differentiated tumour cells, non-CSC tumour populations, or normal tissues. Their expression may vary according to tumour type, disease stage, anatomical location, cellular differentiation state, and treatment history [15–19].

CD133, CD44, and EpCAM illustrate this limitation. Although these molecules have been associated with tumour-initiating or stem-like populations and have been investigated in CAR-based approaches, their expression is heterogeneous and context-dependent [15–19]. Experimental studies targeting EpCAM have demonstrated the potential of adoptive cellular approaches against EpCAM-expressing tumour cells, but variable antigen expression remains an important consideration for therapeutic translation [87].

The available preclinical evidence is not uniformly supportive of these markers as universal CSC targets. Differences in tumour models, methods used to define CSCs, antigen thresholds, and functional assays can produce apparently conflicting results. Consequently, enrichment of an antigen within a CSC population should be distinguished from demonstration that the antigen is necessary for CSC maintenance or that its targeting results in long-term CSC eradication.

### Antigen heterogeneity and dynamic CSC states

Antigen heterogeneity represents a major obstacle to single-antigen CSC targeting. Even when a candidate antigen is strongly enriched within a CSC population, expression may vary between patients and between regions of the same tumour. Furthermore, CSC plasticity permits transitions between stem-like and differentiated states, potentially resulting in acquisition or loss of target-antigen expression during tumour progression or therapeutic pressure [27, 28, 36, 38].

This dynamic behaviour has two major implications. First, a CAR directed against a single CSC-associated antigen may eliminate antigen-positive cells while leaving antigen-low or antigen-negative tumour cells capable of repopulating the tumour. Second, treatment-induced selection may favor cellular states with reduced antigen expression or altered differentiation programmes. These processes provide a biological rationale for dual-target, tandem, and logic-gated CAR architectures designed to broaden recognition and reduce antigen escape [20, 24, 25].

Therefore, future CSC-targeting strategies should increasingly distinguish between static markers and dynamic cellular states. Longitudinal assessment of antigen expression and cellular phenotype may be more informative than measurement at a single pretreatment time point. This distinction also provides a rationale for incorporating single-cell, spatial, transcriptomic, and longitudinal datasets into computational target-prioritization frameworks.

### Normal-tissue expression and on-target/off-tumour toxicity

Safety represents a critical limitation because several candidate CSC-associated antigens are not exclusively restricted to malignant stem-like cells. Expression by normal stem, progenitor, epithelial, endothelial, or other tissues may create the potential for on-target/off-tumour injury when CAR cells recognize the same antigen outside the tumour. Consequently, a biologically attractive CSC marker may not necessarily be an appropriate therapeutic target.

This problem is particularly relevant when antigen expression is widespread or when normal tissues depend on the targeted antigen for physiological maintenance. Therapeutic development must therefore consider not only the degree of tumour or CSC enrichment but also antigen density, tissue distribution, accessibility, and the consequences of eliminating antigen-positive normal cells.

AI-based approaches may assist safety assessment by integrating tumour and normal-tissue expression datasets, identifying candidate antigens with favourable tumour-to-normal expression ratios, and modelling potential receptor–antigen interactions. However, computational predictions cannot independently establish clinical safety. Experimental validation using normal primary cells, organoid systems, tissue panels, and appropriate in vivo models remains necessary before clinical evaluation [27, 28, 36, 38].

### Multi-antigen and context-dependent targeting

The limitations of individual CSC-associated antigens support the development of multi-antigen strategies. Dual-target and tandem CARs can increase the probability of recognizing heterogeneous tumour-cell populations, whereas logic-gated designs can incorporate combinations of positive and negative antigen signals to improve tumour selectivity [20, 24, 25]. These approaches may reduce antigen escape but introduce additional engineering complexity and may affect CAR activation thresholds, persistence, and manufacturing.

Target selection should therefore be considered within the biological context of each malignancy rather than through a universal CSC-marker framework. An antigen with favorable enrichment in one tumour type may have limited specificity or therapeutic value in another. Similarly, an antigen that appears stable in untreated disease may become heterogeneous following chemotherapy, targeted therapy, or CAR-mediated selection.

### Implications for precision CSC-targeted CAR development

These considerations indicate that CSC antigen selection should be treated as a multivariable problem involving tumour specificity, antigen density, spatial distribution, temporal stability, functional relevance, and normal-tissue expression. A candidate antigen should therefore progress through a sequence of biological and translational assessments rather than being selected solely because it is enriched in a CSC population.

The relevant framework can be summarized as:

- CSC enrichment → tumour specificity → spatial and temporal stability → functional relevance → normal-tissue safety → CAR recognition → experimental validation → preclinical efficacy → clinical evaluation.

AI may strengthen this process by integrating multi-omics, single-cell, spatial, proteomic, and clinical datasets to identify candidate targets and predict combinations that are difficult to evaluate using individual datasets. Nevertheless, computational prioritization remains a discovery-stage step unless supported by experimental and clinical validation. This distinction is particularly important because an AI model can identify a statistically strong biomarker without demonstrating that targeting the biomarker will produce selective and long- term CSC eradication.

Accordingly, the most promising future strategy is unlikely to involve identification of a single universal CSC antigen. Instead, precision CSC-directed CAR therapy will require identification of tumour- and state-specific antigen combinations, followed by experimental assessment of their therapeutic window and resistance potential. This provides a direct rationale for applying artificial intelligence to dynamic antigen discovery, multi-omics integration, CAR design, patient stratification, and safety prediction, as discussed in the following section.

## AI-assisted precision immunogenetics

Artificial intelligence (AI) and machine-learning approaches provide opportunities to integrate genomic, transcriptomic, proteomic, single-cell, spatial, and clinical datasets for CSC characterization, biomarker discovery, patient stratification, and CAR engineering [10, 87–91]. In this review, precision immunogenetics refers to the integration of these molecular and clinical features to guide the selection and development of individualized CAR-based therapies.

### AI-assisted CSC antigen discovery and multi-omics integration

AI-based analysis can identify molecular patterns associated with CSC-enriched populations and prioritize candidate therapeutic antigens [10, 87–91]. Integration of single-cell, transcriptomic, proteomic, and spatial datasets may further distinguish heterogeneous tumour-cell states and identify dynamic CSC-associated phenotypes [47]. However, computational enrichment does not establish CSC specificity. Candidate targets require experimental confirmation of tumour heterogeneity, temporal stability, normal-tissue expression, and functional relevance before CAR development.

This distinction is particularly important because CSC states can change during tumour progression and treatment. Therefore, AI may be more useful for identifying dynamic and context-dependent target combinations than for selecting a single universal CSC marker.

### AI-assisted CAR design, patient stratification and safety

AI-assisted structural modelling may support prediction of receptor–antigen interactions and optimization of CAR configurations [68–70]. However, improved computational binding or structural predictions do not necessarily translate into enhanced CAR persistence, cytotoxicity, reduced exhaustion, or *in-vivo* efficacy. Functional and preclinical validation therefore remains essential.

AI can also support patient stratification and toxicity prediction by integrating molecular and clinical characteristics [68–70, 82]. However, retrospective prediction of treatment response should not be interpreted as prospective validation of an AI-guided CAR design. External validation across independent cohorts and prospective assessment of clinical utility are required before such models can be considered clinically reliable.

AI may additionally improve safety assessment by integrating tumour and normal-tissue expression data to prioritize targets with a wider therapeutic window. Nevertheless, computational prediction cannot replace experimental evaluation of normal-tissue expression and on-target/off-tumour toxicity.

### AI applications in next-generation CAR platforms and digital twins

AI-assisted modelling has also been proposed for optimizing CAR-NK cells and other next-generation platforms, including persistence, signalling, and manufacturing characteristics [71, 72]. These applications remain predominantly early-stage, and computational optimization has not yet established long-term CSC-specific clinical benefit.

Digital-twin technology could theoretically integrate longitudinal molecular, imaging, treatment, and clinical data to simulate patient-specific therapeutic responses [73]. However, CSC-directed digital twins remain largely conceptual because CSC plasticity, incomplete longitudinal sampling, parameter uncertainty, and tumour evolution make patient-specific modelling difficult. Mathematical simulation should therefore not be considered equivalent to a clinically validated digital twin.

### Evidence maturity and translational perspective

The evidence supporting AI applications in CSC-targeted CAR therapy varies considerably. Current approaches range from computational proof-of-concept and preclinical validation to retrospective clinical modelling, whereas prospective clinical validation remains limited. These evidence levels should not be considered equivalent.

Accordingly, the translational pathway should progress from “biological rationale → computational discovery → experimental/preclinical validation → clinical testing → prospective AI-guided clinical utility”. AI should currently be regarded primarily as a discovery and decision-support technology rather than an independently validated therapeutic intervention.

The evidence maturity and principal limitations of individual AI applications are summarized in Table 3.

**Table 3:** AI applications in CSC-targeted CAR immunotherapy: evidence maturity and translational status

AI application	AI/computational approach	Potential application	Current evidence/validation stage	Key translational limitation	Representative references
CSC-associated antigen discovery	Machine learning/pattern recognition	Prioritization of candidate CSC-associated targets	Conceptual/computational; selected experimental validation	CSC specificity and normal-tissue expression require experimental confirmation	[91]
Single-cell and multi-omics analysis	Deep learning/multimodal modelling	Identification of CSC-enriched states and tumour heterogeneity	Computational/discovery-stage	CSC rarity, batch effects, annotation variability and limited longitudinal data	[91]
Protein structure prediction	AI-assisted structural modelling	Prediction of receptor–antigen interactions and CAR configurations	Computational; requires functional validation	Predicted binding does not establish cellular efficacy or safety	[68]
CAR construct optimization	Machine learning/computational modelling	Optimization of signalling and engineering parameters	Computational to preclinical	Functional persistence, exhaustion and in-vivo efficacy require validation	[69, 70]
Patient stratification	Multimodal AI	Prediction of treatment response or patient subgroups	Retrospective modelling in relevant applications	External and prospective clinical validation required	[91]
Toxicity prediction	Predictive machine learning	Prediction of treatment-associated toxicity	Computational/retrospective depending on application	Rare toxicities and underrepresented normal tissues may limit prediction	[68]
CAR-NK optimization	AI-guided modelling	Optimization of persistence, signalling and manufacturing	Early computational/preclinical development	CSC-specific clinical benefit remains unestablished	[71, 72]
Digital-twin modelling	Computational simulation/multimodal AI	Patient-specific therapeutic simulation	Predominantly conceptual/computational	Parameter uncertainty, longitudinal data requirements and lack of prospective validation	[73]

Evidence stages are comparative and describe the maturity of the AI application, not the overall maturity of the CAR platform. Computational or retrospective evidence should not be interpreted as prospective clinical validation

## Translational challenges and limitations

Despite rapid advances in CSC biology, CAR engineering, and AI, several barriers continue to limit translation into clinically effective CSC-targeted therapies. These challenges arise from both the underlying biology of CSCs and limitations in the datasets, computational models, and clinical infrastructure required for precision immunotherapy.

### CSC data scarcity and biological heterogeneity

AI models depend on sufficiently large, representative, and well-annotated datasets. However, CSCs constitute a relatively small and heterogeneous fraction of many tumours, and their representation in publicly available datasets is consequently limited [34, 92]. Single-cell and spatial approaches can improve resolution, but differences in tissue processing, sequencing platforms, batch effects, and cell-state annotation can introduce additional variability.

CSC plasticity further complicates model development because stem-like states may emerge or disappear in response to therapeutic pressure [35, 93–96]. Models based on static datasets may therefore fail to capture treatment-induced state transitions and evolving antigen expression. Longitudinal single-cell and spatial datasets will be important for developing models capable of distinguishing stable therapeutic targets from transient CSC-associated states.

### Model bias, data leakage and generalizability

AI performance may be affected by sampling bias, class imbalance, batch effects, incomplete clinical annotation, and underrepresentation of rare CSC populations. Models may also learn dataset-specific technical features rather than biologically meaningful signals. Data leakage between training and validation datasets can further produce overly optimistic performance estimates.

External validation across independent institutions, tumour types, patient populations, and technical platforms is therefore essential. Reported model performance should be interpreted together with cohort composition, validation strategy, calibration, and clinical relevance rather than relying on accuracy or discrimination metrics alone.

### Interpretability and clinical accountability

AI models used for target selection or treatment prediction may produce complex predictions that are difficult to interpret biologically. Explainable AI approaches may help identify molecular features contributing to model predictions and facilitate biological assessment of candidate targets. However, interpretability alone does not establish causality or therapeutic validity.

For clinical implementation, responsibility for AI-assisted decisions must also remain clearly defined. Reproducibility, data governance, patient privacy, model updating, and regulatory oversight will be important as computational systems become integrated into therapeutic decision-making [97].

### Limited prospective clinical validation

A major translational limitation is the gap between computational performance and demonstrated clinical utility. Numerous AI models have been developed using retrospective datasets, whereas relatively few have undergone prospective evaluation [82, 98, 99]. This distinction is particularly important for CSC-targeted CAR therapy, where an algorithm may successfully identify an antigen or predict treatment response without demonstrating that its use changes treatment selection or improves patient outcomes.

Prospective multicenter studies should therefore evaluate AI predictions before treatment and determine whether their use improves clinically meaningful endpoints. External validation should precede widespread clinical implementation.

### Integration into clinical CAR-therapy development

The practical implementation of AI-guided CAR therapy also requires integration across multiple stages, including tumour sampling, molecular profiling, computational analysis, CAR manufacturing, quality control, and clinical monitoring. Differences in laboratory infrastructure, manufacturing capacity, data standards, and clinical workflows may limit implementation even when computational models perform well.

Furthermore, CSC-directed therapy requires simultaneous consideration of target specificity, tumour heterogeneity, CAR-cell function, TME-mediated resistance, and toxicity. AI is therefore unlikely to provide a single solution to CSC resistance. Its greatest value may instead arise from integrating multiple biological and clinical variables to support decisions that remain experimentally and clinically testable.

### Key translational priorities

Future studies should prioritize:

- development of standardized, multicenter CSC-specific datasets;
- longitudinal single-cell and spatial profiling of treatment-resistant tumour;
- identification of dynamic CSC states rather than reliance on single static markers;
- external validation and prospective evaluation of AI models;
- interpretable and reproducible computational workflows;
- experimental validation of AI-prioritized CAR targets and designs;
- integration of TME and metabolic features into therapeutic modelling; and
- clearly defined regulatory and clinical accountability frameworks.

These priorities are important for moving AI-guided CSC-targeted CAR therapy from computational prediction toward reproducible and clinically meaningful application. The principal limitations and corresponding future strategies are summarized in Table 4.

**Table 4:** Current translational challenges and future priorities for AI-guided CSC-targeted CAR immunotherapy

Challenge	Impact on translation	Priority solution
Limited CSC-specific datasets	Reduced model performance	Development of large, multicenter, standardized CSC-specific datasets
CSC heterogeneity and plasticity	Poor model generalizability	Longitudinal single-cell and spatial profiling
Batch effects and sampling bias	Dataset-specific predictions	Data harmonization and independent external validation
Data leakage	Overestimated model performance	Strict separation of training, validation, and test datasets
Limited external validation	Uncertain clinical applicability	Independent multicenter validation across diverse patient cohorts
Poor model interpretability	Limited biological and clinical trust	Development of explainable and reproducible AI approaches
Limited prospective validation	Unproven clinical utility	Prospective clinical studies evaluating AI-guided decisions
Regulatory and implementation barriers	Delayed clinical adoption	Standardized governance, data protection, and clinical accountability frameworks

Successful clinical implementation will require standardized datasets, rigorous external and prospective validation, interpretable models, and appropriate regulatory oversight

*CSC* cancer stem cell, *CAR* chimeric antigen receptor, *AI* artificial intelligence

## Translational roadmap and future priorities

The translation of AI-enabled CSC-targeted CAR therapy into clinical practice requires a stepwise approach that links biological discovery with experimental validation and prospective clinical evaluation. Rather than assuming that computational prediction will directly improve therapeutic outcomes, each stage should generate evidence required for progression to the next.

### From CSC biology to target identification

The first priority is to define CSC-associated states using integrated molecular, cellular, and spatial approaches. Because CSC phenotypes can change during tumour progression and treatment, future studies should focus on dynamic CSC states and clinically relevant antigen combinations rather than relying exclusively on individual static markers.

### Computational discovery and experimental validation

AI can subsequently be used to integrate multi-omics and clinical datasets to prioritize candidate antigens, identify resistant cellular states, and support CAR design. Computational predictions should then undergo experimental validation of antigen expression, tumour specificity, normal-tissue distribution, CAR recognition, cytotoxicity, and potential toxicity before therapeutic development.

### Preclinical optimization of CAR platforms

Preclinical studies should evaluate CAR-T, CAR-NK, CAR-M, gamma-delta CAR-T, and CAR-iNKT platforms according to their biological advantages and limitations rather than assuming equivalent therapeutic maturity. Evaluation should include CSC eradication, tumour infiltration, persistence, exhaustion, interaction with the TME, manufacturing feasibility, and safety.

For solid tumour, future CAR designs may need to combine antigen targeting with strategies that address CAF-mediated exclusion, hypoxia, metabolic stress, immune checkpoints, and other components of the CSC-supportive niche.

### Clinical testing and prospective AI validation

Promising computational and preclinical approaches should progress through appropriately designed early-phase clinical studies before claims of clinical effectiveness are made. AI models used for patient selection, toxicity prediction, or treatment optimization should subsequently be evaluated prospectively, with independent external validation and clinically meaningful endpoints.

### Priority research directions

Key priorities for the field include:

- defining dynamic CSC states using longitudinal single-cell and spatial approaches;
- identifying multi-antigen or logic-gated strategies that reduce antigen escape;
- integrating CSC, CAR-cell, TME, and metabolic features into predictive models;
- developing standardized multicenter datasets with independent validation;
- experimentally validating AI-prioritized targets and CAR designs;
- establishing prospective clinical studies to determine whether AI-guided decisions improve patient outcomes; and
- developing interpretable, reproducible, and regulatory-ready AI workflows.

Together, these priorities provide a translational pathway from biological rationale to computational discovery, experimental and preclinical validation, clinical testing, and prospective AI-guided clinical utility. This framework may help ensure that advances in AI and CAR engineering translate into measurable therapeutic benefit rather than remaining primarily computational or preclinical achievements.

## Conclusion

Cancer stem cells (CSCs) remain an important barrier to long-term chimeric antigen receptor (CAR)-based immunotherapy because of their self-renewal capacity, phenotypic plasticity, antigen heterogeneity, immune evasion, and ability to adapt to therapeutic pressure. Although CAR-T-cell therapy has demonstrated substantial clinical efficacy, particularly in haematological malignancies, effective CSC targeting remains challenging because of CSC-intrinsic resistance, CAR-cell dysfunction, tumour microenvironment (TME) suppression, and metabolic stress. Artificial intelligence (AI) and computational approaches may support antigen prioritization, CAR design, patient stratification, and toxicity assessment by integrating multi-omics, single-cell, spatial, structural, and clinical data. However, the evidence remains heterogeneous, with many applications still at the computational or preclinical stage and retrospective clinical modelling more advanced than prospective AI-guided CAR development. Computational predictions should therefore not be interpreted as evidence of therapeutic efficacy or clinical benefit. Future progress requires a stepwise translational pathway encompassing biological characterization, computational discovery, experimental validation, preclinical testing, and prospective clinical evaluation. Priorities include developing standardized longitudinal CSC-specific datasets, improving external and prospective model validation, integrating single-cell and spatial information, and experimentally validating AI-prioritized CAR targets and constructs. Further research should address dynamic CSC states, multi-antigen targeting, TME-mediated and metabolic resistance, and the potential toxicity of targets shared with normal tissues. Interpretable and regulatory-ready computational workflows will also be essential for clinical translation. Comparative evaluation of CAR-T, CAR-NK, CAR-M, gamma-delta CAR-T, and CAR-iNKT platforms should consider CSC eradication, manufacturing feasibility, safety, and clinical maturity without assuming equivalent therapeutic potential. Overall, AI should currently be regarded as a discovery-enabling and decision-support technology rather than a clinically validated substitute for experimental and clinical evidence. Rigorous biological validation and prospective clinical studies are needed to establish whether AI-guided approaches can improve the safety, durability, and effectiveness of CSC-targeted immunotherapy.

## Data Availability

No datasets were generated or analysed during the current study.
